# Technology Acceptance Among Patients With Hemophilia in Hong Kong and Their Expectations of a Mobile Health App to Promote Self-management: Survey Study

**DOI:** 10.2196/27985

**Published:** 2021-09-09

**Authors:** Yin Ting Cheung, Pok Hong Lam, Teddy Tai-Ning Lam, Henry Hon Wai Lam, Chi Kong Li

**Affiliations:** 1 School of Pharmacy, Faculty of Medicine The Chinese University of Hong Kong Hong Kong SAR Hong Kong; 2 Department of Pediatrics, Faculty of Medicine The Chinese University of Hong Kong Hong Kong SAR Hong Kong; 3 Hong Kong Haemophilia Society Hong Kong SAR Hong Kong; 4 Department of Paediatrics & Adolescent Medicine Hong Kong SAR Hong Kong; 5 Hong Kong Hub of Paediatric Excellence The Chinese University of Hong Kong Hong Kong SAR Hong Kong

**Keywords:** mobile health, mHealth, patients, expectations, hemophilia, chronic diseases, rare diseases, self-management

## Abstract

**Background:**

The lifelong management of hemophilia is demanding and complex. In July 2019, we published a review in the *Journal of Medical Internet Research*, summarizing telehealth interventions that facilitate monitoring of bleeding events and promoting the appropriate use of clotting factors among patients with hemophilia. This work has led to the development of a community program that aims to harness technology to promote self-management among patients with hemophilia in Hong Kong.

**Objective:**

Before the inception of this program, we conducted a cross-sectional survey to evaluate the patients’ level of technology acceptance and identify their expectations of the use of mobile technology for self-management of hemophilia.

**Methods:**

In total, 56 participants (75% adult patients and 25% parents of pediatric patients; 87.5% with moderate to severe disease) were recruited from a local nongovernmental organization that serves patients with hemophilia. They rated their perceived confidence and acceptance in using the new mobile technology (score 1 to 5 for each item, with a higher score indicating better acceptance) using a structured questionnaire (adapted from the Technology Acceptance Model). They also identified the top features that they perceived to be the most important components of a mobile app for the self-management of hemophilia. The Mann–Whitney *U* test was used to compare technology acceptance scores across subgroups of different clinical and socioeconomic characteristics.

**Results:**

In general, the participants considered themselves skilled in using mobile apps (mean 4.3, 95% CI 4.1-4.5). They were willing to learn to use the new mobile app to organize their bleeding records (mean 4.0, 95% CI 3.7-4.3) and to manage their health (mean 4.2, 95% CI 4.1-4.5). Participants who lived in public housing (a surrogate marker for lower socioeconomic status in Hong Kong) reported lower technology acceptance than those who lived in private housing (*P*=.04). The most important features identified by the participants concerned documenting of infusion logs (n=49, 87.5%), bleeding events (n=48, 85.7%), and the secure delivery of the bleeding information to health care professionals (n=40, 71.4%).

**Conclusions:**

It is encouraging to infer that patients with hemophilia in Hong Kong are receptive to the use of mobile health technology. The findings of this survey are applicable in designing the key features of a patient-centered, multimodal program harnessing mobile technology to promote self-management among patients with hemophilia. Future studies should evaluate participants’ acceptability and perceived usability of the mobile app via user metrics and assess clinical and humanistic outcomes of this program.

## Introduction

Hemophilia is a rare X-linked recessive hemorrhagic disorder that affects mostly men. Hemophilia A and B are caused by deficiencies of coagulation factors VIII and IX, respectively [1]. One common severe complication of this congenital disorder is spontaneous and repetitive bleeding, particularly in the synovial joints [1]. Poor management can eventually lead to permanent joint deformity and chronic hemarthropathy, a severe type of arthritis caused by bleeding into the joints. In addition to impaired physical functioning, patients may show reduced psychosocial functioning and occupational outcomes owing to frequent hospitalization and absenteeism from work or school [2].

Currently, coagulation factor replacement therapy is the most common and effective treatment for hemophilia [3]. Patients with moderate to severe hemophilia undergo regular infusion of plasma-derived or recombinant coagulation factors to prevent spontaneous bleeding. In addition, coagulation factor replacement therapy may be administrated in the event of break-through bleeding. The introduction of home infusion therapy has also empowered patients and their families to manage the disease in a more independent manner. However, significant barriers and perceived limitations have led to a lack of adherence to treatment among patients. Patients with a poor perception of the consequences of their illness and of the necessity of treatment may reduce their adherence to prophylactic infusions [4]. Individuals with lifelong chronic conditions may present with anxiety and stress, particularly those with hemophilia, who must acquire considerable knowledge and independent management skills at a young age [5].

To address the needs of patients with hemophilia, platforms that involve various types of technology have been implemented to promote health education and good protective health behavior [6]. In July 2019, we published a review in the *Journal of Medical Internet Research* to summarize the literature on the effectiveness of telehealth interventions in improving health outcomes in patients with hemophilia [7]. This review included 16 trials and observational studies and showed that mobile technology seemed to improve patients’ adherence and accuracy in recording infusion logs and bleeding events. Studies on the provision of disease-related information and practical skills regarding the management of hemophilia yielded promising outcomes, especially among adolescent and young adult patients [7]. Patients generally reported improvements in self-efficacy in managing hemophilia after the implementation of telehealth technology, but the sustainability of the intervention depends largely on its usability and the patients’ receptivity.

Emerging studies in the literature are demonstrating the importance of understanding factors that affect the acceptance of health care technology, especially in patients with chronic diseases [8-12]. The concept of technology acceptance broadly refers to perceived usefulness or user satisfaction with a technology and often encompasses other constructs such as system usability, user feedback, perceived ease of use, attitude toward using, intention to use, and actual usage [8]. Studies have shown that affordability and accessibility are important factors influencing technology acceptance and uptake [9-12]. One Chinese study reported that individuals who were younger and had higher education attainment, higher income, and better family support were more likely to use a smartphone [11]. Other behavioral factors such as technology anxiety, resistance to change, and a lack of trust in the use of devices for self-management are associated with resistance with using mobile health apps among patients with diabetes [12]. It is important to identify these barriers during the preimplementation phase so that users’ acceptance and adoption of mobile health technology can be enhanced to maximize the success of the intervention or program.

Approximately 200 patients in Hong Kong currently have mild to severe hemophilia [13,14]. Since January 2020, a team of patient advocates, clinicians, and academic researchers has obtained sponsorship from a local philanthropic organization to establish a community program for patients with hemophilia in Hong Kong. One goal of this program is to harness mobile technology to (1) promote adherence to prophylactic infusion and recording of bleeding events, (2) facilitate timely sharing of self-documented information with clinicians to formulate or modify the treatment plans, (3) promote knowledge about self-management (eg, infusion techniques), and (4) improve social interaction in the patient community.

Before the inception of this program, we conducted a cross-sectional survey to evaluate the patients’ level of technology acceptance and identify their expectations regarding the use of mobile technology for self-management of hemophilia.

## Methods

Between June and December 2019, participants were recruited using consecutive sampling through the Hong Kong Hemophilia Society, the only active nongovernmental organization in Hong Kong that provides services to patients with hemophilia. All participants had received a diagnosis of hemophilia A or B from a hematologist and were able to read Chinese or English. The pediatric patient (<18 years of age) surveys were completed by their parents. The Chinese University of Hong Kong Survey and Behavioral Research Ethics Committee approved this study before its inception (Ref SBRE-18-052), and written consent was obtained from all participants.

The participants completed a structured questionnaire that comprised 3 sections. The first section collected the participant’s demographic and socioeconomic information, and the second section evaluated his/her level of technology acceptance. Eleven questions were developed on the basis of the Technology Acceptance Model, one of the most widely applied models to describe consumer acceptability of information technology [15]. The model posits that perceived usefulness and perceived ease of use are important factors determining whether a newly introduced technology would be accepted by its potential users [8,15]. The participants rated their perceived confidence and acceptance in using the new mobile technology on a 5-point Likert scale (1=strongly disagree, 5=strongly agree). The score for each item was summed to yield a total score ranging 11-55, with a higher score indicating better acceptance of the technology. In the last section, the participants were asked to identify the top 6 features (from a list of 10) that they perceived to be the most important components of a mobile app for self-management of hemophilia. These are the 10 most common features according to a review of the existing literature on the use of mobile technology among patients with hemophilia [6,7,16-18]. The participants had the option to provide additional factors and justifications. The questionnaire was developed in traditional Chinese and was piloted with 5 patients. The questionnaire was disseminated in both paper-based and electronic formats. Self-administration of the questionnaire required approximately 10 minutes.

Descriptive statistics were used to summarize the data. The Mann–Whitney *U* test was used to identify differences in the technology acceptance score observed across clinically relevant subgroups: disease severity (mild to moderate versus severe), treatment type (prophylaxis versus on-demand therapy), housing type (public versus private housing, which is a surrogate marker for low versus high socioeconomic status, respectively, in Hong Kong), and highest educational attainment (for adult patients only). An exploratory analysis was conducted to evaluate the reliability (Cronbach α) of the scale. All statistical analyses were performed using SAS (version 9.4, The SAS Institute) and the tests were 2-tailed.

## Results

In total, 56 participants completed the study (Table 1) (response rate=100%). Their average age was 37.2 (SD 14.5) years for adult patients (n=42, 75%) and 10.0 (SD 2.8) years for pediatric patients (n=14, 25%). Most of the patients (n=42, 81%) had a diagnosis of hemophilia A with a moderate to severe condition (n=49, 88%). Approximately half of the cohort (n=26, 46%) lived in public housing.

**Table 1:** Demographic and clinical characteristics of respondents (N=56).

Characteristics		Patients (n=56)		Parents who completed the survey on behalf of pediatric patients (n=14)	
		n (%)	mean (SD)	n (%)	mean (SD)
**Age (years)**		—^a^	30.4 (17.4)	—	41.5 (6.6)
	Adult patients	42 (75.0)	37.2 (14.5)		
	Pediatric patients	14 (25.0)	10.0 (2.8)		
**Highest education attainment**			—		—
	Secondary and below	30 (53.6)		10 (71.4)	
	Post-secondary and above	26 (46.4)		4 (28.6)	
**Types of** h**ousing**			—	—	—
	Public	26 (46.4)			
	Private	23 (41.1)			
	Others	7 (12.5)			
**Diagnosis**			—	—	—
	Hemophilia A	42 (75.0)			
	Hemophilia B	10 (17.8)			
	Did not indicate/not sure	4 (7.2)			
**Treatment type**			—	—	—
	Prophylaxis	38 (67.8)			
	On-demand therapy	17 (30.4)			
	Did not indicate/not sure	1 (1.8)			
**Disease severity**			—	—	—
	Mild	4 (7.2)			
	Moderate	16 (28.6)			
	Severe	33 (58.9)			
	Did not indicate/not sure	3 (5.3)			

^a^—: not applicable.

**Figure 1:**
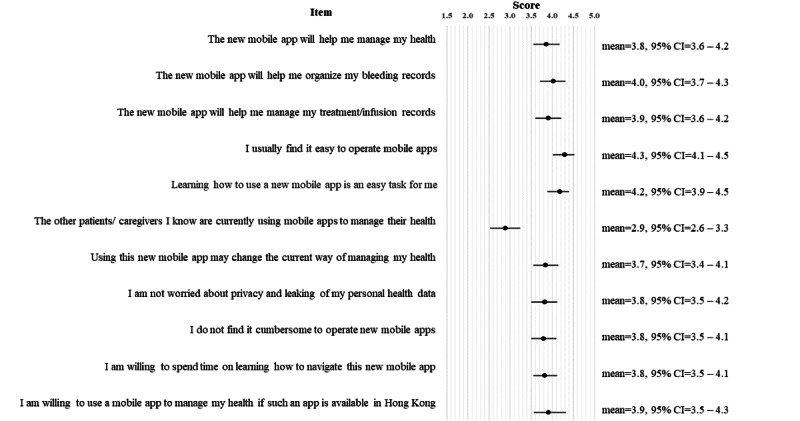
Participants’ level of technology acceptance and confidence in using mobile technology (n=56). Questions were developed on the basis of the Technology Acceptance Model [15]. The participants reported their perceived confidence and acceptance in using the new mobile technology on a 5-point Likert scale (1=strongly disagree, 5=strongly agree). A higher score is indicative of better acceptance of the technology. Error bars represent the 95% CI values.

The mean technology acceptance score was 42.3 (95% CI 40.1-44.4; range 27.0-55.0). In general, the participants considered themselves skilled in using mobile apps (mean 4.3, 95% CI 4.1-4.5; Figure 1). They were willing to learn to use the new mobile app to organize their bleeding records (mean 4.0, 95% CI 3.7-4.3) and to manage their health (mean 4.2, 95% CI 4.1-4.5). Cronbach α values of the scale were .89, .90, and .87 for the overall cohort, adult patients, and parents of pediatric patients, respectively, which indicate high internal consistency.

Participants who lived in public housing (mean 39.9, 95% CI 37.1-42.9) reported lower technology acceptance than those who lived in private housing (mean 44.4, 95% CI 41.3-47.3; *P*=.04). No significant association was identified between the technology acceptance level and disease severity (*P*=.17), treatment type (*P*=.91), or education level (*P*=.75, adult patients only).

**Figure 2:**
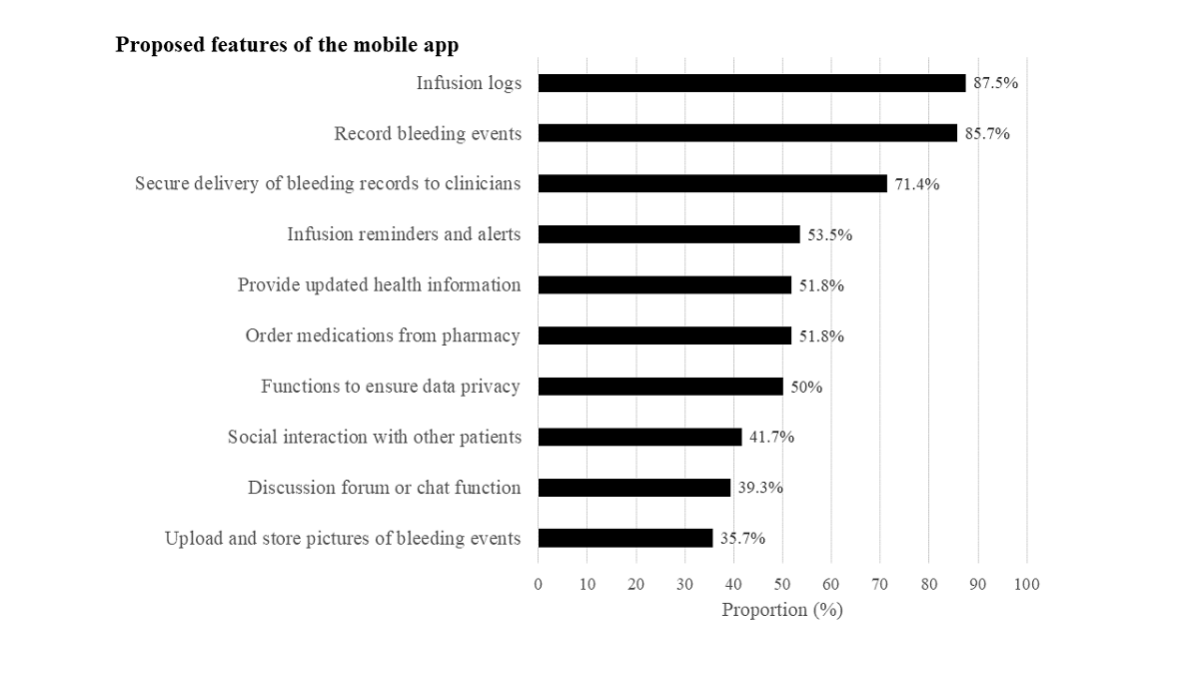
Participants’ preferred features of the mobile technology (n=56). Participants were asked to identify the top 6 features (from a list of 10) that they perceived to be the most important components of a mobile app for self-management of hemophilia. These features were selected on the basis of a review of the existing literature [6,7,16-18].

The most important features identified by the participants concerned documenting of infusion logs (n=49, 88%), bleeding events (n=48, 86%), and secure delivery of the bleeding information to health care professionals (n=40, 71%; Figure 2). One participant proposed health care appointment reminders to be considered as an additional feature as he struggled with “remembering his scheduled appointments with the hematology, physiotherapy, and orthopedics specialists.”

## Discussion

### Principal Findings

This survey aimed to learn about patients’ and parents’ perceptions of adopting mobile technology as an approach to manage hemophilia. We found that a large majority of our prospective users were skilled in using mobile apps. They also expressed confidence and support in learning how to use the new health apps. Participants who lived in public housing demonstrated lower acceptance and perception to the mobile technology compared to those who lived in private housing, concurrent with previous findings [8-12], which have identified lower socioeconomic status as a barrier to the acceptance and adoption of mobile health technology. These subgroups of users may require more preintervention training and regular personal contact during the implementation phase.

We applied the findings of this survey and the literature to design the key features of a patient-centered program harnessing mobile technology. The mobile app will be developed in traditional Chinese, the most common written language in Hong Kong. The first implementation phase will focus on development and promotion of the “documentation” features (ie, recording bleeding events, infusion logs, and infusion reminders). The patients and caregivers will first familiarize themselves with these primary features before expanding to secondary features that include social interaction and education functions. This staggered or waved rollout approach will allow us to identify any problems or windows of opportunity that would facilitate subsequent implementation. Considering that a telehealth intervention should not be administered alone [7,17,18], we will adopt multimodal components to complement the telehealth technology. These components include preintervention training via in-house workshops to enhance the users’ proficiency and engaging patient advocates to champion this intervention. A peer-mentorship program will complement the multimedia educational platforms to enhance knowledge transfer and information utilization. To alleviate the participants’ concerns about privacy and data security, we will implement authentication systems and encryption to protect the patients’ electronic health information. The development and maintenance of the mobile app will be hosted by the information technology service center of an academic institution with well-established cybersecurity systems. In addition, the participants will be assured that only deidentified data will be exported at the back end solely for research and quality improvement purposes.

### Limitations

The findings of this study have to be interpreted with caution owing to a potential selection bias; patients who were interested in the program might be more likely to have participated in this survey than those were not. The small sample size is expected because hemophilia is a rare disease. However, we recruited patients through a nongovernmental organization, and the response rate was high. This approach may have helped to establish the sampling frame and likely reduced the risk of selection bias. Other than conducting an exploratory analysis on the internal consistency of the items, we did not evaluate the other psychometric properties of the survey tool. However, the items were adapted from the Technology Acceptance Model, which is one of the most popular theoretical frameworks among similar studies conducted in other patient populations [19-21]. As this is a preintervention survey, we do expect the users’ perceptions and preference to change after we launch the mobile app. To evaluate the success of this program, our future work will include the collection of data on the participants’ acceptability and perceived usability of the mobile app on the basis of user metrics (number of downloads and installs, acquisition, stickiness, and active users). We will also assess clinical outcomes (adherence to prophylactic treatment and reduction in bleeding events) and humanistic outcomes (users’ satisfaction and health-related quality of life).

### Conclusions

Initiation of this multimodal program that includes mobile technology would be parallel with the vision embodied by “The Smart City Blueprint for Hong Kong” [22], which was introduced by the Government of the Hong Kong SAR to facilitate the use of innovation and technology to improve people’s quality of life. It is encouraging to infer that patients with hemophilia in Hong Kong are receptive to the use of mobile technology as part of a multimodal program to improve self-management of their health. However, like with any service project, there is a risk of failure if the program is not implemented in a thoughtful way. As identified by the study participants, there is a need for ongoing promotion and monitoring of usability, particularly in the early implementation phases. Additionally, continual maintenance, quality assurance, and data security should be ensured to maximize the sustainability of this project.
